# Morbimortalidad perioperatoria de las cirugías cardíacas en el Instituto Nacional Cardiovascular, Lima, Perú, durante el año 2023

**DOI:** 10.47487/apcyccv.v6i1.446

**Published:** 2025-02-12

**Authors:** Harod Silva-Tejada, Josué Sisniegas-Razón, Franklin Martínez-Ninanqui, Zoé Díaz-Chávez, Josías C. Ríos-Ortega

**Affiliations:** 1 Servicio de Cirugía Cardiovascular, Instituto Nacional Cardiovascular, EsSalud, Lima, Perú Servicio de Cirugía Cardiovascular Instituto Nacional Cardiovascular, EsSalud Lima Perú

**Keywords:** Cirugía Cardíaca, Mortalidad, Perú, Cardiac Surgery, Mortality, Peru

## Abstract

**Objetivo.:**

Determinar la mortalidad posoperatoria y las complicaciones posoperatorias de pacientes sometidos a cirugía cardiaca en el Servicio de Cirugía Cardiovascular del Instituto Nacional Cardiovascular, EsSalud, Lima, Perú en el año 2023.

**Materiales y métodos.:**

Estudio descriptivo, retrospectivo, de las historias clínicas de los pacientes.

**Resultados.:**

En el año 2023 se realizaron 538 cirugías cardiacas en nuestro centro, la mortalidad global fue del 5,6%; sin embargo, la mortalidad de cirugías electivas fue del 4,1%. La mortalidad de la cirugía coronaria aislada fue del 1,9% y de la cirugía valvular asilada fue de 0,7%. El *Stroke* se presentó en el 1,5% de pacientes, la reoperación por sangrado se realizó en el 8,1% de los casos. La cirugía más frecuentemente realizada fue la valvular (ya sea aislada o multivalvular) con el 40,1% de los casos, seguida de la coronaria con el 28,6%. La estancia hospitalaria de la cirugía coronaria tuvo una mediana de 9,9 días (8-12) y de la cirugía valvular aislada fue 12,8 días (10-14).

**Conclusiones.:**

Los resultados posoperatorios de la cirugía cardíaca en el Instituto Nacional Cardiovascular son aceptables y comparables a los de otros centros internacionales de alto volumen de cirugías.

## Abstract (en)

**Objective.:**

Determine postoperative mortality and postoperative complications of patients undergoing cardiac surgery in the Cardiovascular Surgery Service of the Instituto Nacional Cardiovascular, EsSalud, Lima, Peru, in the year 2023.

**Materials and Methods.:**

We carried out a descriptive, retrospective study of the patients' medical records.

**Results.:**

In 2023, 538 cardiac surgeries were performed in our center; overall mortality was 5.6%; however, mortality from elective surgeries was 4.1%. The mortality of isolated coronary surgery was 1.9% and of isolated valve surgery was 0.7%. Stroke occurred in 1.5% of patients; re-operation due to bleeding was performed in 8.1% of cases. The most frequently performed surgery was valve surgery (either isolated or multivalvular) with 40.1% of cases, followed by coronary surgery with 28.6%. The hospital stay for coronary surgery had a median of 9.9 days (8-12), and for isolated valve surgery it was 12.8 days (10-14).

**Conclusions.:**

The postoperative results of cardiac surgery at the National Cardiovascular Institute are acceptable and comparable to those of other international high-volume surgical centers.

## Introducción

La enfermedad cardiovascular es la mayor causa de muerte en el mundo, con más de 17,5 millones de muertes anuales a nivel mundial, de los cuales el 75% ocurren en países de ingreso medio-bajo ^1^. Más de 17 millones de personas fallecen de condiciones quirúrgicamente prevenibles cada año, y solo el 6% de los 313 millones de intervenciones quirúrgicas anuales que se hacen a nivel mundial se realizan en poblaciones de bajos recursos, lo que muestra la enorme desigualdad en el acceso al tratamiento oportuno de las enfermedades cardiovasculares ^2^. El Instituto Nacional Cardiovascular (INCOR) es un centro peruano de referencia nacional para pacientes de la seguridad social (EsSalud), donde se tratan pacientes de la capital y provincias de diferentes estratos económico.

Durante la pandemia del COVID -19 el volumen de cirugías cardiacas disminuyó en forma importante según datos internos de la institución. La CDC (Centers for Disease Control and Prevention) demostró que la tasa de mortalidad ajustada a la edad en relación a enfermedad cardiaca se incrementó en el 2020, la más alta desde el 2012, este hallazgo sugiere que la pandemia puede haber impedido que los pacientes más pobres y marginados llegaran a los hospitales, lo que posiblemente provocó que murieran en el hogar debido a síndrome coronario agudo, estenosis aórtica crítica u otras afecciones cardiovasculares tratables ^3^.

En América Latina, el desarrollo de la cirugía cardiovascular tiene como máximo representante a Brasil seguido por México, Argentina y Colombia. Brasil es el único país en Latinoamérica que desde el 2014 ha logrado instaurar un registro de cirugía cardiaca en adultos con una base de datos en funcionamiento que actualmente recopila información de 17 centros participantes en todo el país y está ampliando constantemente el grupo de unidades contribuyentes, el registro nacional BYPASS ^4^.

En Perú, el registro de casos sobre cirugía cardiaca de adultos en los diversos centros es escasa o nula ^5^. El INCOR, fundado en 1992, es el centro de referencia y mayor volumen en cirugía cardiaca del Perú. En el año 2022, se publicó el primer reporte sobre actividad en cirugía cardiaca de la institución con el título «Análisis de las cirugías cardíacas y mortalidad operatoria en el Instituto Nacional Cardiovascular durante el 2022 ^6^ el cual mostró resultados equiparables con centros de referencia. 

El objetivo del presente estudio es describir los resultados de las cirugías cardiacas según patología, así como sus complicaciones y mortalidad a los 30 días del posoperatorio en el INCOR durante el año 2023 y dar de esta manera continuidad al registro del centro.

## Materiales y métodos

### Diseño de estudio

Estudio descriptivo, retrospectivo en el Servicio de Cirugía Cardiovascular del Instituto Nacional Cardiovascular, Lima, Perú. El objetivo principal fue determinar la mortalidad posoperatoria según tipo de cirugía, el objetivo secundario fue determinar las complicaciones posoperatorias (*Stroke*, Infarto de miocardio posoperatorio, bloqueo aurículoventricular) y otras variables clínicas.

### Población de estudio

Historias clínicas de todos los pacientes que ingresaron al Servicio de Cirugía Cardiovascular del Instituto Nacional Cardiovascular de EsSalud, Lima, Perú entre el 1 de enero y el 31 de diciembre del 2023 y que fueron sometidos a cirugía cardíaca.

### Variables

• Mortalidad. Mortalidad por todas las causas en los primeros 30 días del posoperatorio.
• Tipo de cirugía. Cirugía valvular: cirugía que involucró uno más procedimiento en las válvulas cardiacas. Cirugía coronaria: procedimiento de *bypass* coronario aislado con o sin circulación extracorpórea. Cirugía combinada: procedimiento que combinó cirugía valvular y coronaria en el mismo paciente. Cirugía de aorta: cirugía que abarcó diversas enfermedades de la aorta, como disección, úlcera penetrante, hematoma intramural, aneurisma, pseudoaneurisma.
• Complicaciones posoperatorias. Determinadas hasta los primeros 30 días del posoperatorio. *Stroke*: infarto, accidente isquémico transitorio o sangrado cerebral cuyo diagnóstico figure en la historia clínica confirmado por imagen tomográfica y/o resonancia o por evaluación clínica de un neurólogo especialista. Infarto de miocardio posoperatorio: según la cuarta definición de infarto de miocardio ^7^. Bloqueo aurículoventricular: que requirió colocación de marcapasos definitivo. Ventilación mecánica prolongada: intubación orotraqueal durante más de 48 h en el período posoperatorio. Fibrilación auricular (FA): nuevo diagnóstico de FA, paroxística o permanente en el posoperatorio.
• Estancia hospitalaria. Determinada desde el día de la cirugía hasta el alta, en días.

### Aspectos éticos

El presente estudio contó con la aprobación de Comité de Ética e Investigación del Instituto Nacional Cardiovascular (046/2024 CEI -INCOR). Además, se respeta la confidencialidad de los datos.

### Análisis de datos

Las variables cuantitativas son expresadas como media y desviación estándar o mediana y rango intercuartil, según corresponda por criterios de normalidad. Las variables cualitativas se expresan como total y porcentajes. Utilizamos gráficos y tablas de frecuencias. Para la mortalidad y complicaciones posoperatorias se consideró la incidencia acumulada.

## Resultados

**Figura 1:**
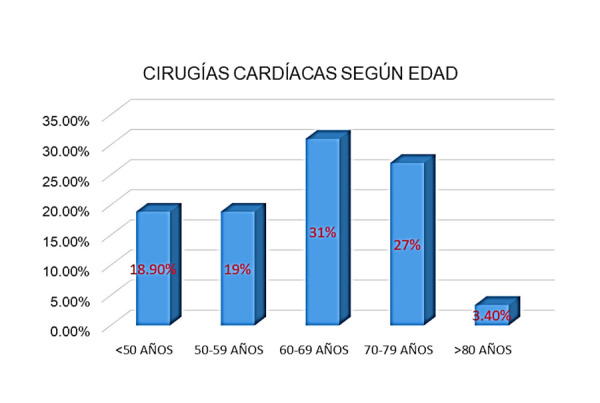
Distribución de las cirugías según edad. Se observa que la mayor población se concentra entre los 60-79 años.

En el año 2023 se realizaron 538 cirugías cardiacas, de las cuales 364 (68%) corresponden a pacientes de sexo masculino. En la Figura 1 podemos observar la distribución de las cirugías por edad, casi el 60% de los pacientes tenían entre 60-79 años.

En la Tabla 1 se muestra la distribución según el tipo de cirugía. Se ve claramente que la cirugía valvular (ya sea aislada o multivalvular) fue la cirugía más realizada con 216 casos (40,1%); la cirugía coronaria fue la segunda cirugía más frecuentemente realizada, con el 28,6% de los casos (154 cirugías). Es de notar que durante todo el 2023 se realizaron siete trasplantes cardíacos y trece pacientes recibieron soporte circulatorio mecánico ya sea con ECMO o con dispositivos de asistencia de larga permanencia. En nuestro centro esta terapia fue usada como puente a decisión o recuperación en pacientes con falla cardiaca por múltiples causas (no solo por choque poscardiotomía).

**Tabla 1:** Tipos de cirugía cardíaca realizadas en el INCOR durante el año 2023

Tipo de cirugía	Mes												Total	%
	Ene	Feb	Mar	Abr	May	Jun	Jul	Ago	Sep	Oct	Nov	Dic				
Cirugía coronaria	14	13	16	11	12	20	19	14	7	8	9	11	154	28,6
Cirugía valvular aislada	18	20	18	9	8	6	5	11	11	13	10	9	138	25,6
Cirugía multivalvular	6	2	12	9	8	5	7	5	3	7	10	4	78	14,5
Cirugía combinada	3	5	2	5	12	4	2	2	3	4	0	1	43	8,0
Cirugía de aorta	4	2	4	4	5	2	1	2	5	1	9	5	44	8,2
Otras con CEC	3	1	1	3	4	2	2	3	0	4	6	1	30	5,6
Cardiopatía congénita	2	2	3	4	3	1	1	1	4	2	2	0	25	4,7
ECMO/Asistencia	0	1	0	0	0	0	1	2	2	2	1	4	13	2,4
Trasplante cardiaco	0	0	0	1	1	0	2	0	3	0	0	0	7	1,3
Complicaciones mecánicas posinfarto	0	0	0	0	1	1	2	0	1	0	1	0	6	1,1

CEC: circulación extracorpórea; ECMO: circulación extracorpórea con membrana oxigenatoria.

El reemplazo de la válvula aórtica aislada o combinada con *bypass* coronario fue la cirugía valvular realizada con más frecuencia, con 127 casos, lo que representa el 23,6% del total de cirugías cardiacas en el año (538); (Tabla 2). Con relación a la cirugía coronaria observamos que la revascularización más frecuente fue la de mamaria interna izquierda a la descendente anterior asociada a vena safena como complemento (84 pacientes, 54,4% del total de cirugías coronarias); empero, el uso de más de dos injertos arteriales (mamaria izquierda + radial o mamaria bilateral) se realizó en 61 pacientes, 39,6% del total de cirugías coronarias (Tabla 2). En la Tabla 3 presentamos los tipos de cirugías cardíacas miniinvasivas realizadas durante el 2023, se realizaron 66 cirugías, lo cual representa el 12,3% del total.

**Tabla 2:** Subtipos de cirugía cardíaca con CEC más frecuentemente realizados en el INCOR durante el año 2023

Tipo de Cirugía	N	%
Cirugía valvular	216	100
Cirugía valvular aislada	138	63,9
Reemplazo valvular aórtico	96	44,4
Reemplazo valvular mitral	22	10,2
Reemplazo valvular pulmonar	2	0,9
Reemplazo valvular tricúspide	5	2,3
Reparación valvular tricúspide	3	1,4
Reparación valvular aórtica	5	2,3
Reparación valvular mitral	5	2,3
Cirugía multivalvular	78	36,1
Cirugía de tres válvulas	12	5,6
Reemplazo de una válvula + una reparación	48	22,2
Reemplazo de dos válvulas	13	6
Reparación de dos válvulas	5	2,3
Cirugía coronaria	154	100
Arteria mamaria + vena safena	84	54,5
Arteria mamaria + arteria radial	45	29,2
Arteria mamaria bilateral	16	10,4
Sólo vena safena	9	5,9
Cirugía combinada	43	100
Reemplazo valvular aórtico + revascularización	31	72,1
Reemplazo valvular mitral + revascularización	5	11,6
Doble reemplazo valvular + revascularización	3	6,7
Reparación valvular mitral + revascularización	4	9,3

CEC: circulación extracorpórea

**Tabla 3:** Cirugía cardiaca mínimamente invasiva en el INCOR durante el año 2023

Tipo de Cirugía	N	%
Total	66	100
Reemplazo valvular aórtico	23	34,8
Miniesternotomía superior	3	4,5
Minitoracotomía anterior derecha	20	30,3
Cirugía valvular mitral por minitoracotomía	17	25,8
Reemplazo de válvula mitral	7	10,6
Reemplazo de valvular mitral + reparación de válvula tricúspide	7	10,6
Reparación de válvula mitral + reparación de válvula tricúspide	2	3
Reparación de válvula mitral	1	1,5
Reemplazo valvular tricúspideo por minitoracotomía	2	3
Atrioseptoplastía por minitoracotomía	14	21,2
Atrioseptoplastía aislada	3	4,5
Atrioseptoplastía + reparación de válvula tricúspide	6	9,1
Atrioseptoplastía + corrección de drenaje venoso anómalo parcial	5	7,6
Exéresis de tumor intracardiaco por minitoracotomía	10	15,2
Mixoma cardiaco	7	10,6
Fibroelastoma	1	1,5
Tumores malignos	2	3

La mortalidad global (Tabla 4) fue de 30 pacientes, lo que constituye el 5,6%. Sin embargo, cuando consideramos solo las cirugías electivas, la mortalidad total fue de 4,1%. La cirugía coronaria tuvo una mortalidad global (electiva + emergencia) del 1,9% en tanto que la cirugía valvular aislada tuvo una mortalidad global del 0,7%.

**Tabla 4:** Mortalidad general según momento quirúrgico y tipo de cirugía cardiaca en el INCOR durante el año 2023

Tipo de cirugía	Electiva		Emergencia		Total casos N	Mortalidad total (%)
	Total N	Mortalidad n (%)	Total N	Mortalidad n (%)				
Cirugía coronaria	127	3 (2,36)	27	0 (0)	154	1,9
Cirugía valvular aislada	138	1(0,7)	0	0	138	0,7
Cirugía multivalvular	74	6(8,1)	4	0(0)	78	7,6
Cirugía combinada	41	2(4,8)	2	0(0)	43	4,6
Cirugía de aorta	36	3(8,3)	8	3(37)	44	13,6
Otras con CEC	24	3(12,5)	6	2(33,3)	30	16,6
Cardiopatía congénita	25	1(4)	0	0	25	4,0
ECMO/Asistencia	0	0(0)	13	3(23)	13	23,0
Trasplante cardiaco	0	0(0)	7	1(14,3)	7	14,2
Complicaciones mecánicas posinfarto	0	0(0)	6	2(33,3)	6	33,3
Total	465	19(4,1)	73	11(15)	538	5,6

CEC: circulación extracorpórea; ECMO: circulación extracorpórea con membrana oxigenatoria.

En cuanto a las complicaciones posoperatorias, observamos que el *Stroke* se presentó en el 1,5% de pacientes, el bloqueo aurículoventricular que requirió marcapasos definitivo tuvo una frecuencia del 1,5% del total de cirugías. La reoperación por sangrado excesivo se tuvo que realizar en 43 pacientes (8,1% del total de las cirugías). En la Tabla 5 se muestran todas las complicaciones observadas. Además, en la Tabla 6 presentamos la estancia hospitalaria y la estancia en la Unidad de Cuidados Intensivos (UCI) según el tipo de cirugías.

**Tabla 5:** Complicaciones posoperatorias

Complicación	N	%
Ventilación mecánica prolongada	18	3,3
*Stroke*	8	1,5
Sangrado posoperatorio excesivo	43	8,1
Reintervención cardiaca	9	1,7
Marcapaso permanente	8	1,5
Fibrilación auricular paroxística	55	10,2
Infarto de miocardio perioperatorio	4	0,7
Mediastinitis	3	0,5
Infección de sitio operatorio	40	7,4

**Tabla 6:** Estancia posoperatoria en la unidad de cuidados intensivos y hospitalaria según tipo de cirugía con CEC en el INCOR durante el año 2023

Tipo de cirugía	Estancia posoperatoria en UCI (días)*	Estancia hospitalaria total (días)*
Revascularización miocárdica	3,13 (2-5)	9,9 (8-12)
Valvular aislada	4,00 (3-6)	12,8 (10-14)
Multivalvular	4,53 (3-6)	14,2 (11-19)
Combinada	5,00 (3-11)	16,4 (14-27)
Aorta	3,81 (3-6)	16,9 (11-20)
Otras cirugías con CEC	3,67 (3-6)	11,4 (9-18)
Trasplante cardiaco	10,00 (9-12)	24,0 (21-27)
Complicaciones mecánicas pos-infarto	4,67 (4-7)	18,0 (16-22)
Cardiopatía congénita	5,45 (3-8)	14,0 (10-21)

CEC: circulación extracorpórea. * Datos expresados en mediana y rango intercuartil.

## Discusión

En el año 2023 se realizaron 538 cirugías cardiacas en nuestro centro, con una mortalidad global del 5,6. El *Stroke* se presentó en el 1,5% de pacientes y la reoperación por sangrado se realizó en el 8,1% de los casos. La cirugía más frecuentemente fue la valvular seguida de la coronaria. La estancia hospitalaria de la cirugía coronaria tuvo una mediana de 9,9 días y de la cirugía valvular aislada fue 12,8 días .

El número total de cirugías realizadas en el 2023 (538 cirugías) supera el número de cirugías reportadas en el 2022 (503 cirugías) en nuestro centro, esto probablemente se deba a una mejor disponibilidad de recursos después de la pandemia ^6^.

La mayoría de pacientes operados fueron varones entre los 60-79 años; con relación a esto, el análisis de los datos del Registro Español de Cirugía Cardiaca 2021-2023 mostró que el 67,7% de los pacientes intervenidos fueron varones, con una media de edad de 68,3 años (DE 12,8) y el 44,1% eran mayores de 70 años ^8^. En el registro de la Sociedad de Cirujanos Torácicos (STS, por sus siglas en inglés) de Norteamérica, se reportó que entre el 2015 al 2022 el promedio de edad de pacientes sometidos a cirugía de *bypass* coronario fue de 65,6 años ^9^. Estos datos son similares a los obtenidos en nuestro centro, y establecen que los varones entre 60-79 años son los que más requieren una intervención al corazón.

En nuestro centro, la cirugía más realizada fue la valvular y, de esta, la cirugía más frecuente fue el cambio de la válvula aórtica, data muy parecida a la reportada en el 2022 ^6^. Sin embargo, estos datos son disímiles a los del Registro del STS que muestran que la cirugía coronaria fue la más frecuentemente realizada en Norteamérica (77% de los casos operados en el 2023); probablemente, esta preponderancia de la cirugía coronaria sobre la valvular se deba a la drástica disminución del remplazo de la válvula aórtica convencional y el aumento sustancial del implante percutáneo de dicha válvula (TAVI) en estos países (USA y Canadá) ^9^. El registro BYPASS de Brasil (17 centros que operan cirugía cardíaca) reportó que entre abril del 2014 a abril del 2018 se operaron 910 pacientes de patología valvular (26% del total de 3500 pacientes), y el reemplazo de la válvula aórtica fue la cirugía valvular más frecuentes (34% de los casos) ^10^. En España, también la cirugía valvular es la más frecuente y representó entre el 52,1 al 57,6% entre el 2021 al 2024 ^8^.

En relación a la mortalidad, nuestra mortalidad global fue 5,6%, ligeramente mayor a la reportada en el 2022 (4,5%) y similar a la observada entre el 2021 al 2023 en España (5,1%)^6^^,^^8^. La mortalidad de la cirugía coronaria fue del 1,9%. El registro del STS reportó una mortalidad promedio de 1,91% entre los pacientes operados de *bypass* coronario entre el 2015 al 2022 ^9^. Sin embargo, en el análisis del registro español se encontró una mortalidad del 3,2% para la cirugía coronaria aislada y en el registro BYPASS de Brasil la mortalidad para la misma cirugía fue de 2,8%. ^8^^,^^11^. Estos datos nos permiten concluir que nuestra institución es un centro de excelencia para realizar la cirugía de *bypass* coronario.

La mortalidad de la cirugía valvular aislada (aórtica o mitral) tuvo una mortalidad del 0,7%. En comparación a esto el registro español encontró una mortalidad del 7,1% para el reemplazo de la válvula mitral y 2,2% para el reemplazo de la válvula aórtica ^8^. Por otro lado, el registro brasileño encontró una mortalidad para el reemplazo de la válvula aórtica y mitral del 5,1 y 5,0%, respectivamente ^10^. La mortalidad más alta observada en nuestra serie dentro de las cirugías electivas es la patologia de aorta, y dentro de las cirugías de emergencia se encuentran las complicaciones mecánicas postinfarto y nuevamente la patología de aorta, en el registro español se encontró una mortalidad del 10% para cirugía de la aorta ^8^.

En relación a las complicaciones, una revisión sistemática de 174 969 pacientes, encontró que la tasa de *stroke* poscirugía cardíaca fue de 0,98% (IC95%: 0,79 - 1,23%) y la tasa de eventos agrupados de mortalidad operatoria fue del 28,8% (IC del 95%: 17,6 a 43,4%) para el *stroke* que se presenta en los primeros días de la cirugía, comparado con 2,4% (IC95%: 1,9-3,1%) en aquellos pacientes sin *Stroke*^12^. Por otro lado, de un total de 10 250 pacientes operados en el Centro Médico de la Universidad de Pittsburg entre 2010 - 2017, la tasa de *Stroke* fue de 2,16%, la mortalidad operatoria fue significativamente mayor para los pacientes que sufrieron un accidente cerebrovascular posoperatorio (14,93% frente a 2,15%, p <0,001). Los predictores de accidente cerebrovascular incluyeron edad avanzada, enfermedad cerebrovascular conocida, diabetes *mellitus* y cirugía de emergencia ^13^. Nuestra tasa de stroke posoperatorio fue del 1,5%, datos comparables con la casuística internacional.

El sangrado posoperatorio que requiere una nueva exploración se asocia con una menor supervivencia a largo plazo y un mayor riesgo de eventos adversos a corto plazo, incluyendo mortalidad operatoria, accidente cerebrovascular, complicaciones renales y respiratorias, y una estancia hospitalaria más prolongada ^14^. La Clínica Mayo reportó una tasa de reoperación por sangrado del 3,3% en pacientes sometidos a cirugía cardíaca entre 1993 al 2019; sin embargo, el registro brasileño BYPASS encontró que la tasa de sangrado mayor en la cirugía valvular fue del 9,9% y para la cirugía de *bypass* coronario fue del 2,7% ^10^^,^^11^^,^^15^.Nuestra tasa para 2023 es del 8,1%, exactamente igual a la reportada el año 2022, las cuales consideramos son significativamente mayores a las de los centros de excelencia, esto debido a causas que no se han podido delucidar, lo cual seria motivo para realizar más estudios.

Una de las complicaciones más frecuentes luego de una cirugía cardíaca es la FA posoperatoria, cuando esta se presenta aumenta considerablemente la estancia hospitalaria. En un estudio retrospectivo en dos centros de Massachusetts en 21 568 pacientes, se encontró que la tasa de FA posquirúrgica fue del 40,8% en mujeres y 38,8% en varones; no obstante, otros autores encuentran tasas entre el 50-60% ^16^^,^^17^. Respecto a esto, encontramos que en nuestra seria la tasa de FA posquirúrgica fue menor (10,2%).

Nuestra estancia hospitalaria es mayor en relación a la reportada por otros centros; por ejemplo, el registro español mostró una estancia posoperatoria global de 8 días (IQR 6 - 13)^8^. Nuestra estancia prolongada quizás se deba a que buena parte de los pacientes son del interior del país y muchas veces no pueden salir de alta por no tener un buen lugar donde continuar el cuidado posoperatorio, sumado al hecho de que muchos otros se quedan internados para optimizar el índice internacional normalizado (INR) antes del alta (como vimos, nuestra principal cirugía es la valvular).

Con relación al uso de dos o más injertos arteriales para la cirugía de *bypass* coronario, en el año 2023 se ha visto un incremento del 27 al 40% en comparación al 2022. En el registro brasileño se observa que solo el 6% de los pacientes recibió arteria mamaria bilateral o mamaria izquierda + radial ^11^. Según el registro del STS, de un total de 281 515 pacientes operados de *bypass* coronario entre el 2018 al 2019, solo 5,6% recibió arteria mamaria bilateral y 8,5% arteria mamaria izquierda + radial ^18^. Los datos de estudios observacionales sugieren que la revascularización con múltiples injertos arteriales puede mejorar la supervivencia a largo plazo en comparación con la derivación coronaria convencional entre un 15 y un 20% ^19^.

En nuestro centro tenemos un programa de cirugía valvular miniinvasiva, en el 2022 se realizaron 49 cirugías cardiacas de mínimo acceso (9,7%), mientras que en el 2023 se ejecutaron 66 (12,3%). Está demostrado claramente que este tipo de cirugías disminuye la estancia hospitalaria, la cantidad de sangrado y el dolor posoperatorio ^6^^,^^20^.

Este estudio tiene algunas limitaciones, pues presenta los resultados de la cirugía cardiaca de un solo lugar. Es un estudio retrospectivo donde los datos recogidos pueden tener errores. Sin embargo, el registro de las historias clínicas en nuestro centro tiene un alto estándar de calidad porque es un centro de referencia nacional. Por otro lado, la publicación de este estudio es de importancia ya que no existe mucha data en nuestro país sobre el tema en mención.

En conclusión, los resultados posoperatorios de la cirugía cardíaca en el Instituto Nacional Cardiovascular en el año 2023 son aceptables y comparables a los de otros centros internacionales de alto volumen de cirugías.

## Fuente de financiamiento:

Autofinanciado
